# Osteosarcoma of the Mandible Masquerading as a Dental Abscess: Report of a Case

**DOI:** 10.1155/2012/635062

**Published:** 2012-11-29

**Authors:** Sukumaran Anil, Anitha P. Krishnan, R. Rajendran

**Affiliations:** ^1^Department of Periodontics and Community Dentistry, College of Dentistry, King Saud University, P.O. Box 60169, Riyadh 11545, Saudi Arabia; ^2^Department of Oral and Maxillofacial Pathology, College of Dentistry, Salman Bin Abdulaziz University, P.O. Box 153, Al-Kharj 11942, Saudi Arabia; ^3^College of Dentistry, King Saud bin Abdulaziz University for Health Sciences, P.O. Box 22490, Riyadh 11426, Saudi Arabia

## Abstract

An aggressive and fatal case of osteosarcoma of the mandible in a 19-year-old female is reported. Six weeks after the clinical appearance of the swelling, the patient died. This paper is unique in that the age of occurrence and the biologic behavior of the tumor were not consistent with the reported literature. The case report is followed by a brief review of osteosarcoma of the jaw with a note on its clinical presentation, diverse radiologic appearance, varied histopathologic picture, and prognosis.

## 1. Introduction

Osteosarcomas are rare malignant neoplasms with a high rate of mortality. They are malignant connective tissue tumors originating from undifferentiated mesenchymal cells that are able to form bone or osteoid tissue [1]. The most common location is the metaphyseal region of long bones, with the area directly above or below the knee accounting for almost half of all cases [2]. Approximately 7% of all osteosarcomas arise in the jawbones [3–5]. The occurrence of osteosarcoma of the jaws is about a decade later than in patients with long bone tumors. The peak age for jaw tumors is 30 to 39 years. Males slightly outnumber females in reported cases [1, 4, 6].

Maxilla and mandible are affected equally, with males showing a predilection for occurrence in the mandible and females in the maxilla [6, 7]. Mandibular lesions are located in the body, symphysis, angle, or ramus in descending order of frequency, while maxillary lesions most often involve the alveolar ridge, antrum, sinus floor, and palate [8, 9]. Clinical symptoms include pain, swelling, loose teeth, separation of teeth, and paresthesia [10].

Radiographically, osteosarcoma can appear with a variable bone density depending on the amount of bone formed by the neoplasm. In some cases, the typical “sunray” appearance is observed at the periphery of the tumor. The changes may be very subtle and difficult to recognize in the early course of the disease. Widened periodontal ligament space has been described as a classical sign of early osteosarcoma [11]. Computed tomography and magnetic resonance imaging are valuable adjuncts in evaluating the extent of the tumor and its relationship with neighboring tissues [12].

Histologically, osteosarcomas are composed of malignant spindle cells which produce foci of osteoid or immature bone. In the jaws, about half of the lesions demonstrate a cartilaginous differentiation [13]. In general, osteosarcomas of the jaws tend to be better differentiated than their long bone counterparts, with some tumors exhibiting a deceptively bland histological appearance [14, 15]. Therefore, correlation of the histological features with the clinical and radiographic findings is essential for the diagnosis.

## 2. Case Report

**Figure 1:**
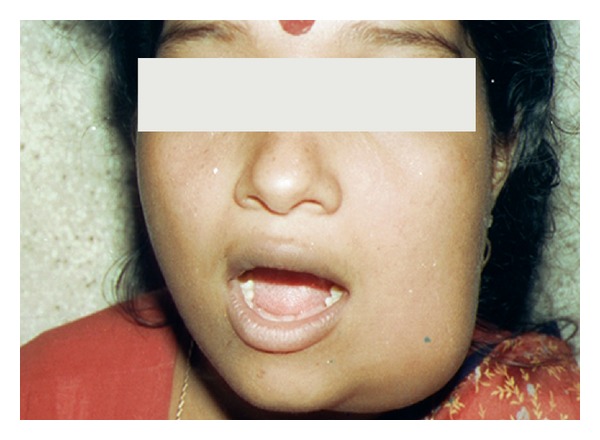
Clinical appearance of the swelling showing the diffuse borders.

A 19-year-old, single, female was referred to the Dental College and Hospital, University of Kerala, India, by a general dental practitioner after treating her in vain with antibiotics for dental abscess for a period of ten days. The patient presented with a complaint of a diffuse swelling on the left side of the mandible, large enough to cause her aesthetic anxiety. She had mild tenderness on palpation of the swelling and slight discomfort in the last molar region of the affected side while chewing. History revealed nothing of significance. At the time of clinical examination, the swelling was of two weeks duration. On examination, the swelling was circumscribed, bony hard, and roughly about 6 × 5 cm in size at the angle of the mandible. The swelling showed diffuse borders. The skin overlying the swelling was of normal color but had a glossy appearance, probably due to tautness of the skin over the swelling (Figure 1). Intraorally, the swelling was evident on the mandibular buccal vestibule adjacent to the second molar. There was apparent expansion of the buccal cortical plate. Lingual cortical plate also showed expansion but to a lesser extent. The mandibular third molar was missing, and the patient explained that both her mandibular wisdom teeth were extracted due to recurrent infection a couple of years ago.

Premolars and the first molar on the affected side were healthy with no carious or periodontal involvement. The second molar showed grade I mobility. Bidigital palpation did not reveal any lymph node enlargement anywhere in the cervicofacial chain.

**Figure 2:**
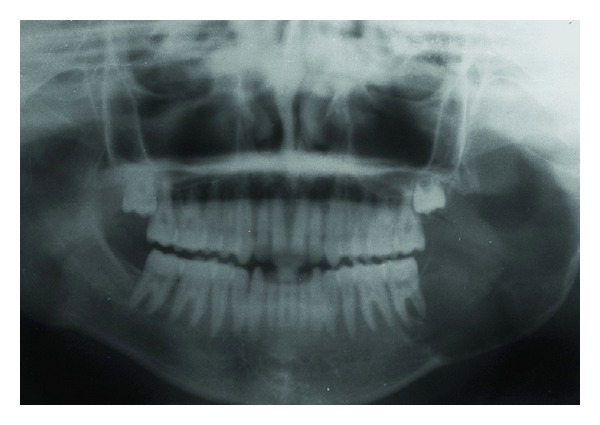
Panoramic radiograph showing the tumor at the left ramus of the mandible.

**Figure 3:**
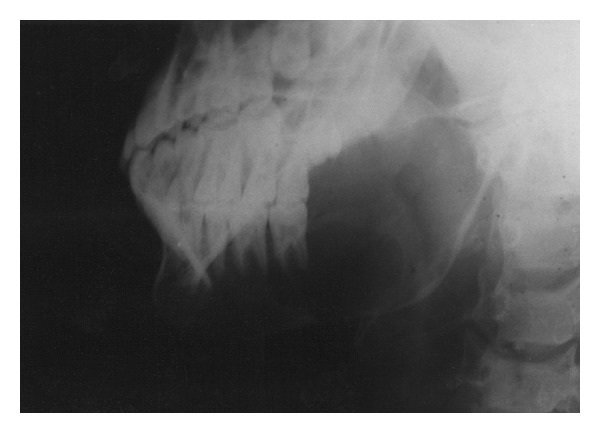
Lateral oblique view of the mandible showing the extent of the tumor.

Panoramic and lateral oblique views of the mandible were ordered. The orthopantogram showed a large radiolucency at the angle of the mandible on the left side, involving the second molar (Figure 2). The tooth appeared to float in space with bony attachment apparent only mesially. The circumscribed cortical plate expansion showed a centrifugal growth pattern involving the angle and major part of ramus of the mandible (Figure 3). The maxillary third molar of the affected side was present within the bone, but the mandibular third molar was absent corroborating the history and clinical finding. There was no widening of the periodontal ligament space and there was no sign of periodontal bone loss anywhere else. The inferior margin of the body of the mandible on the affected side had a moth eaten appearance in the lateral oblique view and a discontinuity in the inferior border suggestive of a pathologic fracture was apparent at the junction of the body and ramus.

**Figure 4:**
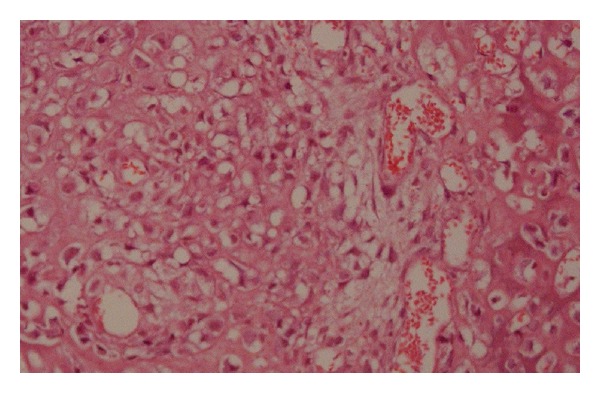
Showing proliferating spindle and oval-shaped mesenchymal cells with tumor osteoid and tumor bone formation HE ×96.

An incision biopsy was performed. The histopathologic picture showed tissue lined with stratified squamous epithelium. Numerous proliferating spindle and oval-shaped mesenchymal cells with tumor osteoid and tumor bone formation were strewn subepithalially. Some areas showed highly pleomorphic cells with hyperchromatic nucleus and bizarre nuclear-cytoplasmic ratio with numerous vascular channels (Figure 4). Without much difficulty a diagnosis of osteosarcoma, osteoblastic variant, of the mandible was arrived.

Patient was recalled and interrogated for any paresthesia or numbness over the affected area. She admitted to a tingling sensation which had been present for a long time. Since it had not caused her any discomfort, she deemed it irrelevant to be mentioned. The serum alkaline phosphatase level was within normal limits. The patient was referred to the Regional Cancer Center for expert management. We followed up the patient's progress and learned that her CT scan did not show any metastatic lesion in the body and also her nuclear bone scan showed an increased isotope uptake at the lesional site. The patient was planned for a radical hemi-mandibulectomy, but before the scheduled date she died. Death was due to massive uncontrolled local disease. It had been a mere six weeks from the time she reported to a dental clinic with the complaint of swelling to her demise. At the time of her death, the swelling on her jaw had doubled in size and the skin over the swelling was stretched tighter and had a deep bluish hue to it.

## 3. Discussion

The clinical presentation of the case was classic with minimal symptoms for such a large swelling and involving posterior mandible. But the rapid rate of growth of the lesion from the time the patient was seen initially to the time of her death was not consistent with the general growth pattern of jaw osteosarcomas. Osteosarcomas of the jaws are usually biologically distinct from those of long bones in that they behave better than their long bone counterparts [16, 17].

The numbness due to compression or infiltration of the inferior alveolar nerve in the mandibular canal of the affected area has been well documented in osteosarcoma and could be an indication of poor prognosis. In this case, even though numbness was not a presenting complaint of the patient, she was having the tingling sensation much before the swelling become apparent. Numbness or paresthesia of the affected area as an early diagnostic feature of osteosarcoma is thus highlighted by our case [18]. The dark hue over the rapidly growing swelling that was noticed towards the end of her days is attributed probably to telangiectasia of superficial vessels due to tumor compression.

The radiographic picture of the case did not show any widening of the periodontal ligament space of either the affected or the adjacent teeth. The circumscribed bilateral centrifugal cortical plate expansion could mimic a central benign neoplasm, although ossifying fibroma is a slow growing benign lesion and does not cause numbness of the affected area or pathological fracture of the bone. There was loss of lamina dura of both the mesial and distal roots of the second molar associated with the bone swelling. This could also be found in periodontal infections, odontogenic cysts and tumors, metastatic tumors, and so forth. Even though the patient had numbness of the affected area, radiographic feature of mandibular canal involvement was not evident. Probably, the extensive periostitis reaction masked this finding [10]. The characteristic sunray appearance and Codman's triangle seen in osteosarcoma of the long bones is less commonly encountered in jaw lesions [2]. The difficulty in diagnosing osteosarcomas by radiological means is mainly because of its wider spectrum of variable.

Rise in serum alkaline phosphatase is reported in osteosarcoma but is not considered to be a consistent finding [19]. However, the present case did not show an elevation in serum alkaline phosphatase. The short clinical course of the tumor could be an explanation for this finding.

Differentiation of osteosarcoma from other bony lesions like Paget's disease, fibrous dysplasia, multiple myeloma, and metastatic tumors is based more on microscopical than radiological evidence [19]. Even then different levels of cell differentiation may create difficulty in distinguishing this tumor from reactive stromal proliferations such as fractured callus or from cellular forms of Paget's disease [20].

Besides the size of the lesion, the histologic type and grading of the tumor are important factors in determining the prognosis. Conventional osteosarcoma can be subdivided into osteoblastic, chondroblastic, and fibroblastic histologic variants depending on the extracellular matrix produced by the tumor cells [1].

Other histologic variants include the myxomatous type, telangiectatic type, small cell osteosarcoma, giant cell osteosarcoma, giant cell predominant osteosarcoma, large cell type, fibrous histiocytoma-like type, and epithelioid osteosarcoma [16]. Histologic picture of the present case showed areas of osteoblastic differentiation with some foci of highly pleomorphic cells and cellular anaplasia. This type has a poorer prognosis when compared with the chondroblastic variety. In fact, in our opinion, the histological grading is more important than all the other features combined in determining the prognosis.

The prognosis of osteosarcoma of the jaws is better than that of long bone, with a 5-year-survival rate of 25.8 percent for the maxilla and 34.8 percent for the mandible. The median survival time for the maxilla is 2.9 years and 6.5 years for the mandible [21]. The progress of the neoplastic growth in this case was so rapid that in contrast to the literature, the time span between diagnosis and death was barely six weeks. Probably the swelling had been present for a longer time but went unnoticed by the patient due to lack of any appreciable clinical symptoms. The patient is aware that its presence began only when it grew large enough to be of aesthetic concern. Usually, the prognosis of a jaw osteosarcoma gets better as the age at which it occurs increases [6]. Older patients are reported to have increased resistance to the tumor, thus increasing the chances of a better prognosis [10]. In our case, the patient was younger than the mean age of occurrence for jaw osteosarcomas. Also, the tumor ran a very aggressive biologic course, a feature uncommon for gnathic lesions [16].

Local recurrence and metastasis occur frequently in patients with osteosarcoma. Rate of metastasis in jaw osteosarcomas is lower than those of the long bones [6, 22]. But metastasis is relatively higher in case of postradiation osteosarcoma of the jaws [23]. Metastasis is usually via the bloodstream and in most cases occurs within 2-3 years. The absence of any metastatic spread seen in this case could be attributed to the very rapid rate of growth of the tumor and short clinical course.

Osteosarcomas of the jaws are cytologically unremarkable. Hence, it is important to separate them from benign or reactive lesions like fibrous dysplasia and osteoblastoma, [24]. It is crucial to completely investigate the lesion and arrive at accurate diagnosis in the initial biopsy. To assist in the identification of malignancy besides histopathological examination, special investigations have been carried out. Bone matrix protein is suggested to assist in recognizing malignant osteoid [25]. Osteocalcin is a bone specific protein that may be useful in differentiating osteosarcoma from malignant fibrous histiocytoma [26].

Early diagnosis and complete tumor resection are the most important factors in increasing prognosis of jaw osteosarcomas [27]. Treatment of osteosarcoma is radical surgery [28]. This usually is accompanied by radiotherapy or chemotherapy. Anatomic limitations in the orofacial region cause difficulties in achieving uninvolved margins and for this reason local recurrence of the lesions is high between 33% and 39%. Tumor-free margins in surgery, chemotherapy with multidrugs, and radiotherapy after surgery have effects in the prognosis of osteosarcoma [29].

## 4. Conclusion

The case reported here emphasizes the importance of histopathology in the diagnosis and predicting prognosis of osteosarcomas of the jaws. Eliciting simple but important clinical symptoms like numbness or paresthesia of the affected area is highlighted. X-ray, CT scan, and MRI imaging along with bone scan are valuable adjuncts to microscopy in the early diagnosis and staging of this malignancy.
